# Author Index

**DOI:** 10.1093/bioinformatics/bts795

**Published:** 2012-09-03

Ananiadou, S. i575

Ashkenazy, H. i389

## 

Bérard, S. i382

Bajic, V.B. i444

Basler, G. i502

Battke, F. i542

Baudot, A. i451

Bell, M.J. i562

Ben-Hur, A. i416

Benes, V. i333

Benz, C. i640

Bhardwaj, N. i431

Boerries, M. i495

Boussau, B. i382

Bresler, M. i311

Bucher, P. i395

Buettner, F. i626

Burstein, D. i389

Busch, H. i495

## 

Cao, D. i370

Carbonell, J. i466

Chan, A.H. i311

Chartier, M. i423

Chin, F.Y.L. i356

Chitale, M. i444

Cho, H.-C. i575

Cohen, O. i389

Coin, L.J.M. i370

Collisson, E.A. i640

Cui, Y. i370

## 

Daubin, V. i382

De Moor, B. i569

Dimitrieva, S. i395

Dingler, C. i535

Doak, T.G. i363

Durand, D. i409

## 

Ferreira, E.C. i515

## 

Gallien, C. i382

Ganguly, A. i549

Gao, X. i444

Gaudreault, F. i423

Gillespie, C.S. i562

Glaab, E. i451

Glauche, I. i556

Goldberg, T. i458

Goldstein, T. i640

Gonzalez-Perez, A. i640

Goto, S. i522 i611

Grimbs, S. i502

## 

Hamp, T. i458

Hatsuda, H. i633

Haubrock, M. i509

Haussler, D. i640

Herberg, M. i556

Holm, L. i438

Hua, X. i509

Huang, H. i619

Humphreys, P. i556

## 

Iacucci, E. i569

## 

Jäger, G. i542

Jimenez, C.R. i596

Jin, X. i370

## 

Källberg, M. i431

Kalkan, T. i556

Kihara, D. i444

Kim, S. i619

Klann, M. i549

Klein-Seetharaman, J. i466

Koeppl, H. i549

Korbel, J.O. i333

Koskinen, J.P. i438

Kotera, M. i611

Kowar, S. i495

Koyutürk, M. i349

Kramer, A. i535

Krasnogor, N. i451

Kreutz, C. i529

Kshirsagar, M. i466

Kwon, M.-S. i582

## 

LaFramboise, T. i349

Lai, H. i409

Langlois, R. i431

Larhlimi, A. i502

Lee, S. i582

Lengauer, T. i589

Leung, H.C.M. i356

Li, J. i509

Li, L. i375

Li, Y. i370

Liu, Y. i318

Lopez-Bigas, N. i640

Lord, P. i562

Lu, H. i431

## 

Machado, D. i515

Mahmud, M.P. i325

Messih, M.A. i444

Michaels, R.P. i480

Milenković, T. i480

Minhas, F.u.A.A. i416

Miwa, M. i575

Mizutani, S. i522

Moreau, Y. i569

## 

Najmanovich, R. i423

Nascimento, J.M. i495

Ng, S. i640

Nho, K. i619

Nie, F. i619

Nieselt, K. i542

Nikoloski, Z. i502

Nishimura, Y. i611

## 

Oh, J.M. i582

Ohta, T. i575

## 

Park, T. i582

Parthasarathy, S. i473

Patil, K.R. i515

Pauwels, E. i487 i522

Pavlopoulos, G.A. i569

Pfeifer, N. i589

Pham, T.V. i596

Popovic, D. i569

Pupko, T. i389

Pyysalo, S. i575

## 

Radde, N. i535

Raue, A. i529

Rausch, T. i333

Ray, S. i349

Ren, J. i370

Rho, M. i363

Risacher, S.L. i619

Rocha, I. i515

Roeder, I. i556

Rost, B. i458

Roth, A.C. i340

Ruffalo, M. i349

## 

Sankoff, D. i402

Sathaye, D. i409

Saykin, A.J. i619

Schelker, M. i529

Scherf, N. i556

Schlattl, A. i333

Schliep, A. i325

Schmidt, B. i318

Schneider, R. i451 i569

Selbig, J. i502

Sheehan, S. i311

Shen, L. i619

Shih, Y.-K. i473

Singh, A. i495

Smith, A. i556

Sokolov, A. i640

Solava, R.W. i480

Song, Y.S. i311

Soons, Z. i515

Stütz, A.M. i333

Stolzer, M. i409

Stoven, V. i487 i522

Stuart, J.M. i640

Sun, L. i370

Swan, D. i562

Sykacek, P. i603

Szöllősi, G.J. i382

## 

Tabei, Y. i487

Takarabe, M. i611

Takemoto, K. i487

Tannier, E. i382

Theis, F.J. i626

Thierbach, K. i556

Timmer, J. i529

Tranchevent, L.-C. i569

Tsujii, J. i575

## 

Valencia, A. i451

Vernot, B. i409

## 

Wang, H. i619

Wang, J. i370 i509

Wang, Y. i356

Weber, P. i535

Wiedenhoeft, J. i325

Wingender, E. i509

Wu, Y.-W. i363

## 

Xu, M. i409

## 

Yamanishi, Y. i487 i522 i611

Yan, J. i619

Yang, S. i370

Ye, Y. i363

Yiu, S.M. i356

## 

Zerjatke, T. i556

Zhang, X. i370

Zheng, C. i402

Zhou, H. i375

Zhu, H. i375

Zichner, T. i333

Zuo, X. i370

